# Cause of Death Among Patients With Diabetes and Heart Failure With Reduced Ejection Fraction

**DOI:** 10.1016/j.jacasi.2022.05.002

**Published:** 2022-10-18

**Authors:** Abhinav Sharma, Carolyn S.P. Lam, Wan Ting Tay, Jonathan Yap, Michael R. MacDonald, Amir Razaghizad, Lauren B. Cooper, Christopher O’Connor, David J. Whellan, Inder S. Anand, Jasper Tromp, Robert J. Mentz

In heart failure (HF), diabetes increases the risk of mortality and HF hospitalization. Despite emerging interest in the area of HF and diabetes, cause-specific death in this population has not been extensively explored. To address this knowledge gap, we evaluated the distribution of cause-specific death in patients with HF with reduced ejection fraction (HFrEF) and diabetes as well as the interaction between diabetes, cause-specific cardiovascular (CV) death, and ethnicity.

Data are from the HF-ACTION (Heart Failure: A Controlled Trial Investigating Outcomes of Exercise Training) trial^1^ and the ASIAN-HF (Asian Sudden Cardiac Death in Heart Failure) registry.^2^ The studies, respectively, recruited 2,331 (left ventricular ejection fraction <35%) and 5,276 patients (left ventricular ejection fraction <40%) across 128 centers. Multivariable models adjusted for age, sex, ethnicity, body mass index, systolic blood pressure, heart rate, New York Heart Association functional class, coronary artery disease, atrial fibrillation, peripheral vascular disease, chronic kidney disease, angiotensin-converting enzyme inhibitors, angiotensin-receptor blockers, beta blockers, diuretic agents, and the competing risk of death from other causes evaluated the association between diabetes and cause-specific death. Interaction analyses were adjusted only for age to prevent overfitting. All events were adjudicated,^1^ and statistical analyses were performed with STATA/SE version 14.0 (StataCorp). The protocols for HF-ACTION and ASIAN-HF were approved by each institution’s and coordination center’s Institutional Review Board. All patients signed a written informed consent form.

**Figure 1:**
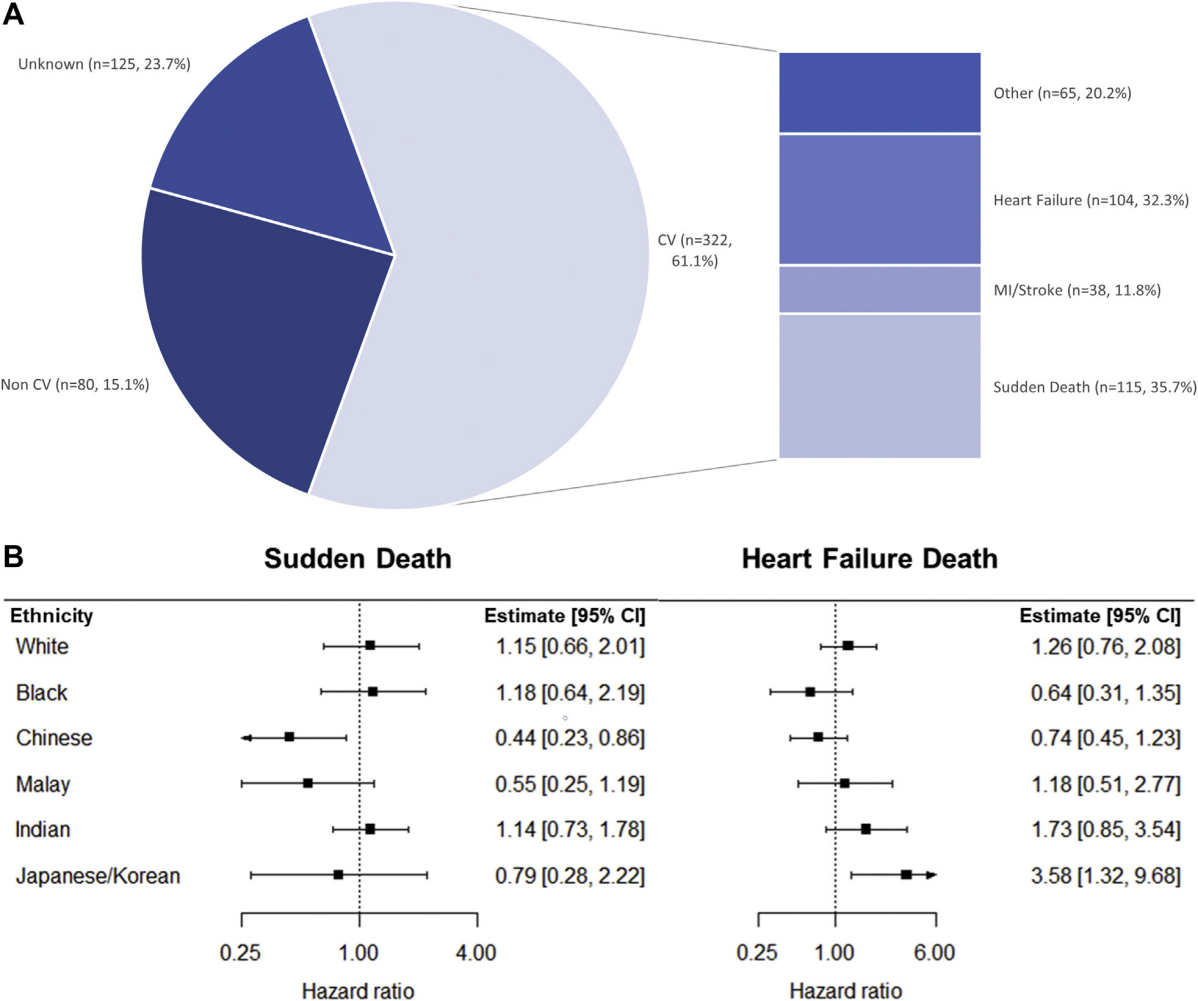
Cause-Specific Death in Diabetes and Heart Failure With Ethnic Variations **(A)** Distribution of cause-specific death in people with diabetes and heart failure with reduced ejection fraction (HFrEF). **(B)** HRs for the risk of cause-specific death in people with HFrEF with vs without diabetes. The findings demonstrate the most common forms of mortality in people with diabetes and HFrEF with ethnic variations derived from Fine-Gray models adjusted for age, sex, and the competing risk of all-cause death. CV = cardiovascular; MI = myocardial infarction.

Together*,* HF-ACTION and ASIAN-HF enrolled 6,182 patients. Among patients with diabetes and HFrEF (n = 2,445), the median age was 61.1 years, 21.3% were female, and 62.6% had an ischemic etiology of HF. There were 527 deaths; 322 (61.1%) were CV, 80 (15.1%) were non-CV, and 125 (23.7%) were unknown. Sudden death was the most common cause of CV death (35.7%; n = 115), followed by HF death (32.3%; n = 104), fatal myocardial infraction/stroke (11.8%, n = 38), and “other” CV death (20.2%; n = 65) (Figure 1A).

In an unadjusted model, diabetes was associated with an increased risk of CV death. However, the significant association disappeared after multivariable adjustment (adjusted HR [aHR]: 1.13; 95% CI: 0.90-1.43). For CV death, there was no interaction between diabetes status, cohort, or ethnicity (all interaction *P* > 0.10). Diabetes was not associated with an increased likelihood of sudden death (aHR: 0.87; 95% CI: 0.60-1.27) or HF death (aHR: 1.08; 95% CI: 0.67-1.59), but it was associated with an increased risk of fatal myocardial infraction/stroke (aHR: 2.29; 95% CI: 1.31-4.0). In interaction analyses, patients with diabetes and ischemic HF vs nonischemic HF had an increased likelihood of CV death (HR: 1.47; 95% CI: 1.21-1.78 vs HR: 1.04; 95% CI: 0.8-1.35; interaction *P* = 0.02).

Ethnicity significantly modified the relationship between diabetes and cause-specific CV death (all interaction *P* < 0.05) (Figure 1B). Chinese patients with vs without diabetes were less likely to die of sudden death (HR: 0.44; 95% CI: 0.23-0.86), whereas Japanese/Korean patients with vs without diabetes were more likely to die of HF death (HR: 3.58; 95% CI: 1.32-9.68).

This study evaluated the association between diabetes and cause-specific death among patients with HFrEF. Our findings showed that in diabetes and HFrEF, CV death and sudden death are the most common causes of mortality and that diabetes does not independently increase the risk of cause-specific CV death. In addition, ethnic variations with respect to cause-specific CV death were shown to exist.

Our findings are consistent with trial data from NAVIGATOR (Nateglinide and Valsartan in Impaired Glucose Tolerance Outcomes Research) and TECOS (Trial Evaluating Cardiovascular Outcomes with Sitagliptin). The NAVIGATOR trial (N = 9,306) recorded 622 deaths, of which 50.3% were non-CV, 39.2% (n = 244) were CV, and 10.1% (n = 65) were unknown.^3^ Similarly, the Trial Evaluating Cardiovascular Outcomes with Sitagliptin trial (N = 14,671) recorded 1,084 deaths, of which 49% (n = 530) were CV, 31% (n = 338) were non-CV, and 20% (n = 216) were unknown.^4^ Our analysis adds to these findings by demonstrating the presence of ethnic variations with respect to cause-specific CV death. This finding is likely explained by the fact that ethnic variations related to patient characteristics, comorbidity prevalence, and health care delivery exist in Asia among patients with HFrEF.^5^^,^^6^

This study is subject to several limitations. Unmeasured confounders (eg, socioeconomic status, regional differences) may have influenced the results, and the proportion of females enrolled may not be representative. There were also differences for the definitions of cause-specific death in HF-ACTION and ASIAN-HF. Finally, we did not adjust for multiple testing, and given the smaller sample size, potential differences in the ethnic risk of cause-specific death may have arisen by chance. However, the well-characterized cohort from 2 major studies with adjudicated causes of death strengthens this analysis.

Among patients with established HFrEF and diabetes, sudden death followed by HF death are the most common causes of CV death. In addition, ethnic variations among patients with and without diabetes were observed regarding the risk of cause-specific CV mortality. Future studies focusing on the prevention of sudden death and HF death should be prioritized among patients with HFrEF and diabetes.
